# A Smart Nano‐Theranostic Platform Based on Dual‐microRNAs Guided Self‐Feedback Tetrahedral Entropy‐Driven DNA Circuit

**DOI:** 10.1002/advs.202301814

**Published:** 2023-04-21

**Authors:** Sha Yang, Jie Luo, Ligai Zhang, Liu Feng, Yuan He, Xueping Gao, Shuang Xie, Mingxuan Gao, Dan Luo, Kai Chang, Ming Chen

**Affiliations:** ^1^ Department of Clinical Laboratory Medicine Southwest Hospital Third Military Medical University (Army Medical University) 30 Gaotanyan, Shapingba District Chongqing 400038 P. R. China; ^2^ Department of Biological and Environmental Engineering Cornell University Ithaca NY 14853‐5701 USA; ^3^ College of Pharmacy and Laboratory Medicine Third Military Medical University (Army Medical University) 30 Gaotanyan, Shapingba District Chongqing 400038 P. R. China; ^4^ State Key Laboratory of Trauma Burn and Combined Injury Third Military Medical University (Army Medical University) 30 Gaotanyan, Shapingba District Chongqing 400038 P. R. China

**Keywords:** dual‐microRNAs, synergistic regulation, tetrahedral entropy‐driven DNA circuit, theranostics

## Abstract

MicroRNAs (miRNAs) can act as oncogenes or tumor suppressors, capable of up or down‐regulating gene expression during tumorigenesis; they are diagnostic biomarkers or therapeutic targets for tumors. To detect low abundance of intracellular oncogenic miRNAs (onco‐miRNAs) and realize synergistic gene therapy of onco‐miRNAs and tumor suppressors, a smart nano‐theranostic platform based on dual‐miRNAs guided self‐feedback tetrahedral entropy‐driven DNA circuit is created. The platform as a delivery vehicle is a DNA tetrahedral framework, in which the entropy‐driven DNA circuit achieves a dual‐miRNAs guided self‐feedback, between an in situ amplification of the onco‐miRNAs and activation of suppressor miRNAs release. To test this platform, dual‐miRNAs are selected, miRNA‐155, an up‐regulated miRNA, as cancer indicators, and miRNA‐122, a down‐regulated miRNA as therapy targets in hepatocellular carcinoma, respectively. Through the circuit, the platform to detect onco‐miRNAs at femtomolar level as well as visualized miRNAs inside cells, fixed tissues, and mice is programmed. Furthermore, triggered by miRNA‐155, preloaded miRNA‐122 is amplified via the self‐feedback and released into target cells; the sudden increase of miRNA‐122 and simultaneous decrease of miRNA‐155 synergistically served as therapeutic drugs for gene regulation with enhanced antitumor efficacy and superior biosafety. It is envisioned that this nano‐theranostic platform will initiate an essential step toward tumor theranostics in personalized/precise medicine.

## Graphical abstract

A smart nano‐theranostic platform based on dual‐miRNAs guided self‐feedback tetrahedral entropy‐driven DNA circuit is created to detect low abundance of intracellular oncogenic miRNAs (onco‐miRNAs) and to realize synergistic gene therapy of onco‐miRNAs and tumor suppressor miRNAs.

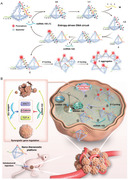

## 1 Introduction

MicroRNAs (miRNAs), a key regulator in post‐transcriptional gene expression, have been demonstrated as oncogenes or tumor suppressors with an up or down expression in tumorigenesis and progression;^[^ ^1^, ^2^, ^3^ ^]^ they are becoming important diagnostic, prognostic, and predictive biomarkers,^[^ ^4^, ^5^, ^6^ ^]^ and therapeutic targets.^[^ ^7^, ^8^, ^9^ ^]^ On the one hand, as potential biomarkers, oncogenic miRNAs (onco‐miRNAs) broadly exist in various body fluids exhibiting specific expression patterns corresponding to tumor status, for example, size and grade.^[^ ^10^ ^]^ And inhibition of over‐expressed onco‐miRNAs can generate effective tumor therapy.^[^ ^11^ ^]^ On the other hand, as novel therapeutics, tumor suppressor miRNAs are great targets to treat post‐transcriptional dysregulations of signaling pathways caused by genetic and epigenetic alterations.^[^ ^12^ ^]^ Different tumor‐related miRNAs affect tumor progresses through different signaling pathways, including oncogenic promotion of tumorgenesis and tumor suppression with opposite roles.^[^ ^13^, ^14^ ^]^ By targeting these miRNAs with opposite roles (raising tumor suppressors and reducing onco‐miRNAs), we can realize treating tumors synergistically via dual‐directional regulation.

Thus far, only a few investigations have been reported on the miRNA‐based, integration of detection with therapy.^[^ ^15^, ^16^, ^17^ ^]^ For example, Dai et al. reported polydopamine‐coated gold nanorods for miRNA detection and photodynamic therapy.^[^ ^18^ ^]^ Cao et al. developed a dual‐modal therapy with miRNA detection and chemo‐photothermal treatment.^[^ ^19^ ^]^ While these two works realized in principle cancer‐targeted theranostics, the detection limit was only nanomolar (nM) level, which needs to be improved significantly for clinical applications. The therapeutic efficacy was not satisfied either with photothermal therapy, due to its poor tissue penetration and low photothermal conversion efficiency. And the biosafety was limited by thermal damage to surrounding normal tissue and prolonged stay in the body.

To improve the detection limit of intracellular miRNA biosensing, we adopted the entropy‐driven DNA circuit from DNA nanoassembly technology. This circuit in essence is a programmed nucleic acid self‐assembly circuit that is thermodynamically driven forward by the systematic entropic gaining during DNA hybridization.^[^ ^20^, ^21^ ^]^ It is an isothermal amplification that does not need enzymes and does not alter any covalent bonds inside cells. To be more specific, through elaborately programming the three‐stranded substrate complex (S) and fuel strand (F), a target (T) can initiate a self‐feedback cascade chain displacement reaction of S and T to yield waste chains (W).^[^ ^22^, ^23^ ^]^ The circuit can promote its cycling‐recycling with the hybridization‐strand and replacement‐dissociation circuit. Such a nucleic acid circuit can realize signal amplification at the polynomial and even the exponential scale, making it ideal for ultrasensitive detection.^[^ ^24^, ^25^, ^26^ ^]^ Notably, the waste chains released from the above circuits can be fully exploited as a therapeutic agent targeting gene regulation. Hence, the precise control from the amplification of onco‐miRNAs to the release of suppressor miRNAs via the smart‐programmed entropy‐driven DNA circuit can provide a cutting‐edge approach for integrated theranostics.

**Figure 1:**
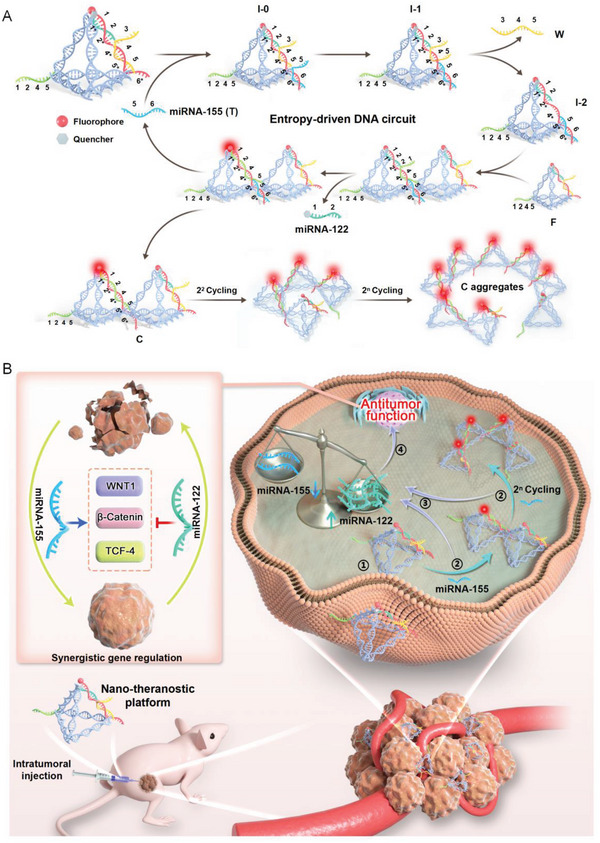
Schematic illustration of a smart nano‐theranostic platform based on dual‐miRNAs guided self‐feedback tetrahedral entropy‐driven DNA circuit. A) The tetrahedral entropy‐driven DNA circuit for fluorescent detection of miRNA‐155 and release of miRNA‐122. B) Simultaneous miRNA‐155 detection and synergistic antitumor function after intratumoral injection of the nano‐theranostic platform. ① Endocytosis. ② Detection of miRNA‐155. ③ Release of miRNA‐122. ④ Synergistic antitumor function. Inset: Synergistic gene regulation of tumor suppressor miRNA‐122 and onco‐miRNA‐155 targeting the Wnt/*β*‐catenin‐TCF signaling pathway. Unbalanced scale: increased miRNA‐122 and decreased miRNA‐155.

To load and subsequently transport this entropy‐driven DNA circuit into living cancer cells, a vehicle is needed; this vehicle should have desirable features, including biostability, biosafety, biocompatibility, the capacity of penetration through biological barriers, and high efficiency of cellular uptake.^[^ ^27^, ^28^ ^]^ Compared with most present intracellular drug delivery vehicles,^[^ ^29^ ^]^ for example, liposomes,^[^ ^30^, ^31^ ^]^ synthetic nanoparticles,^[^ ^32^, ^33^ ^]^ protamine,^[^ ^34^, ^35^ ^]^ PEG,^[^ ^36^ ^]^ and PEI,^[^ ^37^, ^38^ ^]^ the DNA tetrahedron, a simple and representative pyramid DNA nanostructure, has been demonstrated as an effective drug delivery vehicle, especially for nucleic acids.^[^ ^39^, ^40^ ^]^ The DNA tetrahedron is the only delivery vehicle that can load miRNAs directly into its stable DNA structure with all‐natural biocompatibility and high transfection efficiency. The elaborate design of four vertexes and six edges of the DNA tetrahedron can provide opportunities for molecular coupling reactions and spatial transformation, favoring the computation of Boolean logic operations.^[^ ^41^, ^42^, ^43^ ^]^ Consequently, we utilized the tetrahedron as the transmembrane vehicle to directly load and deliver this DNA circuit into cancer cells as a nano‐theranostic platform, for intracellular amplification circuit and activation of tumor suppressors release (**Figure** **1**A,B).

Here, we selected hepatocellular carcinoma (HCC) as the model for our platform, in which dual miRNAs with opposite roles, that is, oncogenic miRNA‐155 and tumor suppressor miRNA‐122, functioned as synergistic regulators (Figure 1B).^[^ ^14^, ^44^, ^45^ ^]^ MiRNA‐155 is markedly up‐regulated in HCC, even in the early stages of HCC, promoting hepatocyte proliferation and tumorigenesis. Thus, miRNA‐155 can serve as a novel, non‐invasive biomarker for early diagnosis, assessment, and prognosis of HCC.^[^ ^46^, ^47^, ^48^, ^49^, ^50^ ^]^ And inhibition of over‐expressed miRNA‐155 can also generate effective tumor therapy.^[^ ^11^, ^51^ ^]^ On the contrary, miRNA‐122, as a hepatocyte‐specific miRNA, is markedly down‐regulated in HCC. Upregulation of miRNA‐122 can suppress HCC growth by inhibiting multiple targets in Wnt/IGF/VEGF signaling pathways.^[^ ^52^, ^53^, ^54^, ^55^ ^]^ Hence, miRNA‐122 can be a treatment drug that suppresses cell proliferation and induces cell apoptosis for HCC.^[^ ^56^ ^]^ Our nano‐theranostic platform for HCC utilized one side of the DNA tetrahedron as S and the opposite vertex as F. Please note that the tumor suppressor miRNA‐122 was preloaded in the S. Once delivered inside HCC cells, the recognition of the target oncogenic miRNA‐155 (T) by entropy‐driven DNA circuit enabled the separation of the fluorophore from the quencher, restoring and amplifying the fluorescence signal. The above process functioned as the detection step. Simultaneously, through self‐feedback, the preloaded tumor suppressor miRNA‐122 was released and miRNA‐155 was reduced within the same cells, functioning as the therapy step. Based on this working mechanism, this nano‐theranostic platform was capable of monitoring onco‐miRNAs in multiple practical settings from liquid, cell, tissue to animal imaging with excellent performance in sensitivity, specificity, stability, and duplication. Meanwhile, this smart platform exhibited promising synergistic therapeutic effects for tumor treatment in vitro and in vivo, and encouraging achievements in tissue penetration and biosafety.

## 2 Results and Discussions

### 2.1 Construction and Characterization of the Nano‐Theranostic Platform

**Figure 2:**
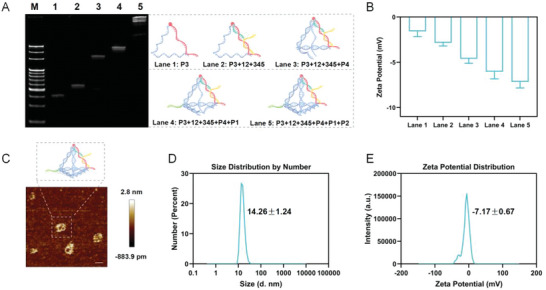
Construction and characterization of the nano‐theranostic platform. A) Native PAGE analysis of the platform self‐assembly. M, 20 bp DNA ladder. B) Zeta potential changes of the assembly process (*n* = 3). C) AFM image of the platform. Scale bar, 10 nm. D) Diameter size and zeta potential of the platform via DLS.

Our nano‐theranostic platform comprised of a DNA tetrahedron serving as a delivery vehicle and the entropy‐driven DNA circuit (one side as the S and one vertex as the F, Figure S1, Supporting Information) functioning as a theranostic tool. The nano‐theranostic platform was constructed by using six specifically‐designed sequences through a continuous annealing process (**Figure** **2**A and Table S1, Supporting Information). The two strands P5 (12) and P6 (345) were hybridized to the corresponding portion (1*2*4*5*) of the strand P3, forming the S. The partial sequences of P3 and the other strands (P1, P2, and P4) formed the tetrahedral framework. When required, the fluorophore and the quencher were labeled on the adjacent locations of P3 and P5 respectively to monitor entropy‐driven DNA circuits initiated by the target.

The construction process of the nano‐theranostic platform was demonstrated by using a 5% native polyacrylamide gel electrophoresis (PAGE, Figure 2A). With new strands added, the electrophoretic mobility decreased due to the increasing of molecular mass and the larger spatial structure. And the zeta potential shifted toward a more negative value during the assembly process, demonstrating the successful construction of the platform (Figure 2B and Figure S2, Supporting Information). Further, atomic force microscopy (AFM, Figure 2C) demonstrated that the shape of the platform was a rigid tetrahedral structure. Dynamic light scattering (DLS, Figure 2D,E) was also performed to confirm that the nano‐theranostic platform was negatively charged particles (−7.17 ± 0.67 mV) with an average diameter size of 14.26 ± 1.24 nm.

To evaluate the storage stability, the nano‐theranostic platforms were stored at 4 °C for different periods, and their integrities were assessed by PAGE (Figure S3, Supporting Information). The grayscale statistics remained stable for the first 4 days and began to degrade in the next 3 days, indicating that our platform had good stability during storage. To test the stability in vitro, the platform was incubated in physiological conditions at 37 °C with 10% fetal bovine serum (FBS) and was tested for fluorescence intensities at different time points (0–12 h). The fluorescence intensities remained unchanged within 8 h and gradually increased (Figure S4, Supporting Information), suggesting that the nano‐theranostic platforms were stable in FBS for 8 h.

### 2.2 Entropy‐Driven Mechanism and Thermodynamics Calculations of the Circuit

Our smart platform was achieved by using the entropy‐driven DNA circuit, comprising of a DNA tetrahedron acting as both S and F. Without the target miRNA‐155, the platform remained intact. Upon exposure to a specific target, the platform was separated into the Intermediate I‐2 sequence and W sequence (Figure 1A). Furthermore, once bonded with F of the tetrahedron, Intermediate I‐2 was promptly dissociated into three separated strands: preloaded miRNA‐122 for therapy, tetrahedral complexes (C) for fluorescent signal generation, and the released target (T) for initiating the next circuit.^[^ ^57^ ^]^ Through multiple circuits, larger C aggregates were produced and enriched, thus causing the amplification of fluorescent signals which had been blocked by the quencher.

**Figure 3:**
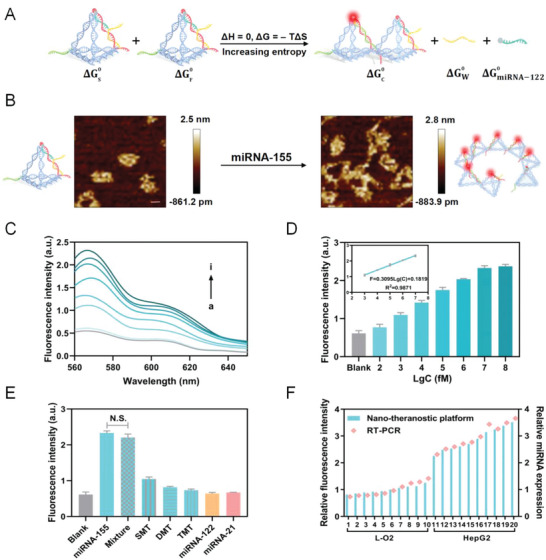
Feasibility of the nano‐theranostic platform for miRNA‐155 detection. A) Reaction equation of entropy‐driven DNA circuit with thermodynamic parameters. B) AFM images without and with the addition of miRNA‐155. Scale bar, 10 nm. C) Fluorescence spectra with response to different concentrations of miRNA‐155 from 560 to 650 nm. a‐i: Blank, 100 fM, 1 pM, 10 pM, 100 pM, 1 nM, 10 nM, and 100 nM. D) Histograms of fluorescence intensities toward different miRNA‐155 concentrations (*n* = 3), data are presented as mean ± SD. Inset: The linear relationship between fluorescence intensities and the logarithmic values of the miRNA‐155 concentrations. E) Histograms of fluorescence intensities toward different other miRNAs (SMT, DMT, TMT, miRNA‐122, and miRNA‐21), and mixture (*n* = 3), data are presented as mean ± SD, and significance is determined using one‐way analysis of variance (ANOVA) with post hoc multiple comparisons (least significant difference, LSD). N.S., not significant. F) Comparison between our platform and RT‐PCR toward miRNA‐155 detection in HepG2 cells and L‐O2 cells.

The reaction equation of the whole DNA circuit was displayed in **Figure** **3**A. The Gibbs free energy was expressed via thermodynamic calculation as Equation (1).

(1) ΔG=ΔH−TΔS

Δ*H*, Δ*S*, and *T* denote systematic enthalpy changes, systematic entropy changes, and thermodynamic temperature, respectively. The numbers of base pairs remained unchanged in the whole circuit, obtaining Δ*H* = 0. Therefore, *T*Δ*S* is the driving force originating from released molecules in this increasing entropy circuit. Consequently, the Gibbs free energy was calculated as eq2.

(2) $$\Delta G=\Delta G_{W}^{0}+\Delta G_{C}^{0}+\Delta G_{\mathrm{miRNA}-122}^{0}-\Delta G_{S}^{0}-\Delta G_{F}^{0}+\mathrm{RT}\ln Q$$

*Q* denotes the reaction quotient, and Δ*G*0 *x* (kcal mol^−1^) refers to the standard free energy of component *x*, which can be obtained via the software NUPACK as follows.

(3) $$\begin{array}{c} \Delta G_{W}^{0}+\Delta G_{C}^{0}+\Delta G_{\mathrm{miRNA}-122}^{0}-\Delta G_{S}^{0}-\Delta G_{F}^{0}=-1.33\mathrm{kcal}\cdot \mathrm{mo}l^{-1} \end{array}$$

When the reaction reached an equilibrium, giving Δ*G* = 0, *Q* was calculated to be 9.11 (combining Equations (2) and (3)). To be more specific, here, the initial concentrations of S and F were both 10 nM. Ultimately, the final concentration of miRNA‐122 (*x* nM) was calculated as below.

(4) $$\frac{{(10^{-9}x)}^{3}}{{[10^{-9}\left(10-x\right)]}^{2}}=9.11$$

Consequently, ***x*** can be estimated between 9.999 and 9.9999 nM in virtue of the bisection method, indicating that the reaction efficiency is 99.99% without taking into account reaction time. Notably, AFM was utilized to confirm the feasibility of this circuit (Figure 3B). Without the target, the nano‐theranostic platform presented a separate pyramid DNA nanostructure. Upon addition of the target, this circuit was triggered, and multiple tetrahedra were interlinked to form C aggregates after several circuit regenerations. This again demonstrated the successful formation of the DNA tetrahedron and regeneratable DNA circuit.

### 2.3 Analytical Performance of the Nano‐Theranostic Platform In Vitro

Afterward, we measured the analytical performance of our nano‐theranostic platform, especially for its sensitivity, specificity, duplication, etc. We primarily optimized the performance of the platform to achieve a high signal‐to‐noise ratio (*F*‐*F*0)/*F*0. After routine optimization, the reaction time, reaction temperature, and Mg^2+^ concentration were determined to be 1 h, 37 °C, and 2 mm, respectively (Figure S5A–C and Table S2, Supporting Information). In addition, the lengths of toehold domains 4 and 6 affect the entropy‐driven kinetics, thus affecting the efficiency of the detection.^[^ ^20^ ^]^ We optimized the lengths of the domains between 4 and 10 bp (Figure S5D and Table S2, Supporting Information). For the subsequent experiments, we chose 4 and 8 bp for toehold domains 4 and 6, respectively.

To evaluate the sensitivity of detecting miRNA‐155, we monitored in vitro the fluorescence intensity changes after the addition of miRNA‐155 at various concentrations (0–100 nM) under the optimal conditions (Figure 3C). As expected, with the concentration increasing, the fluorescence intensity was enhanced. A linear relationship was found between the fluorescence intensity (F) and the logarithmic value of the target concentration LgC; a linear equation of *F* = 0.3095LgC + 0.1819 (*R* ^2^ = 0.9871) was fitted with the range from 1 pM to 10 nM, spanning five orders of magnitude (Figure 3D). The detection limit was calculated to be 114.62 fM based on the rule of 3*σ*/slope; this detection limit is superior or at least equal to those of other intracellular miRNA assay methods (Table S3, Supporting Information). Thus, the detection range and the detection limit of our platform can satisfy the clinical requirements for early diagnosis in vivo. Such an ultrasensitive detection is attributed to the signal amplification via an intrinsic entropy‐driven DNA circuit. Also, the relative standard deviation of the miRNA detection ranged from 0.88% to 11.08% (Table S4, Supporting Information), indicating the duplication of our nano‐theranostic platform.

Specificity is also an important parameter that has been assessed. We generated a variety of control targets including mismatched targets based on binding free energy changes via the NUPACK (single‐, double‐, and triple‐base mismatched targets, SMT, DMT, and TMT, respectively), unrelated targets (other types of miRNAs, i.e., miRNA‐122 and miRNA‐21), and the mixed targets (a 1:1:1 mixture of miRNA‐122, miRNA‐21, and miRNA‐155). The fluorescence intensities were quantified after adding control targets (Figure 3E and Table S5, Supporting Information). The fluorescent signals induced by mismatched targets and unrelated miRNAs are not prominent compared to those from the blank controls. Conversely, the presence of the target, either miRNA‐155 itself or the mixed targets, caused significant signal enhancement with no significant differences between miRNA‐155 and the mixed targets (*p* > 0.05). These results indicated that our nano‐theranostic platform exhibited high specificity for miRNA detection.

To verify that our platform can detect miRNA‐155 in clinical samples extracted the total RNA samples from human hepatoma cell lines (HepG2) and human normal hepatic cell lines (L‐O2, as a control). The expression level of miRNA was then quantified. The same samples were also subjected to the reverse transcription‐polymerase chain reaction (RT‐PCR), which has been viewed as the gold standard for miRNA detection (Figure 3F and Figure S6, Supporting Information). The results from both the platform and the RT‐PCR matched very well (*R* ^2^ of 0.9771 for HepG2, and *R* ^2^ of 0.962 for L‐O2), suggesting the practical feasibility of our nano‐theranostic platform.

### 2.4 Analytical Performance of the Nano‐Theranostic Platform In Vivo

So far, we have used our platform to detect miRNA in vitro and in total RNA extracts from cultured cell lines. To further explore the feasibility in real living samples, we employed confocal laser scanning microscopy (CLSM), fluorescence in situ hybridization (FISH), and the time‐dependent whole body fluorescence scan to explore the in vivo miRNA detection. Our in vivo samples included living cells, in situ hybridization of pathologic tissue biopsies, and living animals (mice).

**Figure 4:**
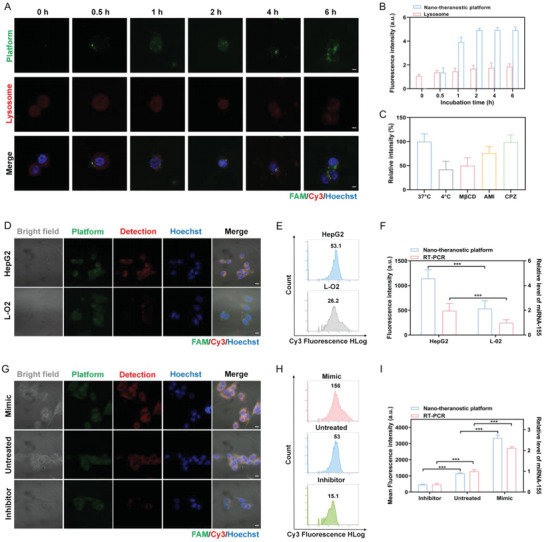
Feasibility of the nano‐theranostic platform for miRNA‐155 detection inside living cells. A,B) Co‐localization of our platforms (green) and lysosomes (red) in cells, and quantitative analysis of time‐evolution fluorescence intensity (*n* = 3), data are presented as mean ± SD. C) Fluorescence intensity of the internalized platforms by HepG2 cells incubated at 4 or 37 °C, or with three endocytosis inhibitors (*n* = 3), data are presented as mean ± SD. M*β*CD: methyl‐*β*‐cyclodextrin. AMI: amiloride. CPZ: chlorpromazine. D–F) CLSM, FCM, and RT‐PCR for miRNA‐155 detection of HepG2 cells and L‐O2 cells (*n* = 3), data are presented as mean ± SD, and significance is determined using independent‐samples T‐tests. G–I) CLSM, FCM, and RT‐PCR for miRNA‐155 detection of up‐regulated, untreated, and down‐regulated HepG2 cells (*n* = 3), data are presented as mean ± SD, and significance is determined using ANOVA with post hoc multiple comparisons (LSD). Scale bars, 10 µm. ***p < 0.001.

Before the formal experiment, we probed the cellular uptake mechanism of our platform (labeled with FAM, green) when internalized into HepG2 cells. We incubated HepG2 cells with the platform at the temperature of 4 and 37 °C, or pretreated with three endocytosis inhibitors (**Figure** **4**C). The cellular uptake at 4 °C was much lower than that at 37 °C, monitored by the decreased fluorescent signal of flow cytometry (FCM), suggesting that the platform was delivered via energy‐dependent endocytosis. Of three endocytosis inhibitors utilized, methyl‐*β*‐cyclodextrin (M*β*CD) and amiloride (AMI) inhibited the platform uptake, manifesting that the majority of our platform was internalized by caveolin‐dependent endocytosis and a small part was endocytosed by macropinocytosis pathways. We went on to perform time‐evolution colocalization experiments, the platforms labeled with FAM fluorophores, and the HepG2 cells stained with red lysotracker for labeling and tracking lysosomes (Figure 4A,B). The platforms entered into the cytoplasm after incubation for 0.5 h and mainly stayed in the cytoplasm with incubation from 1 to 6 h. And few yellow areas, overlapping of green and red fluorescence, started to be observed at 6 h, indicating that only small amounts of the platforms reached the lysosomes.

For miRNA detection in living cells, we monitored the fluorescence of our nano‐theranostic platform incubated with living cells via CLSM. Three distinct dyes (FAM, Cy3, and Hoechst) were employed, FAM (green) was labeled on the strand P2 for accurate positioning of the platform, and Cy3 (orange) was marked on the P3 part of S for precise monitoring of entropy‐driven DNA circuits initiated by miRNA‐155, and Hoechst (blue) for nuclei. With the incubation time within 2 h, the fluorescence intensities drastically increased due to the exposure of intrinsic target miRNA‐155 and initiation of the circuit amplification within the living cells (Figure S8A,B, Supporting Information). Fluorescence intensities stabilized from 2 to 6 h. After incubation for 6 h, the intensities continued to increase, mainly due to the degradation of the platform by cytosolic nucleases, as previously mentioned. These results revealed that the nano‐theranostic platform can remain fully intact for 6 h inside cells, suggesting good biocompatibility and biostability. And 2 h was considered as the incubation time for the subsequent experiments. Afterward, the miRNA‐155 expression profiles in HepG2 cells and L‐O2 cells were detected by utilizing CLSM, and were simultaneously subjected to the FCM and RT‐PCR for validation. It revealed a stronger Cy3 fluorescent signal for HepG2 cells than that for L‐O2 (2.13‐fold, Figure 4D). Similarly, mean Cy3 fluorescence in HepG2 cells monitored by FCM was 2.02‐fold in comparison with that of L‐O2 cells, demonstrating the platform yielded consistent results (Figure 4E). And we further verified the reliability of our platform by the RT‐PCR results, well‐accepted reliable benchmarks. The two results were in good agreement (1.98‐fold, Figure 4F). Likewise, the up‐regulated and down‐regulated miRNA‐155 (transfected with miRNA‐155 mimic and miRNA‐155 inhibitor, respectively) expression levels were monitored by the platform, FCM, and RT‐PCR. In contrast with Cy3 fluorescent intensities of the untreated group (CLSM, Figure 4G), HepG2 cells treated with up‐regulated miRNA‐155 showed a higher signal intensity (2.91‐fold), and the opposite phenomenon was observed with negligible Cy3 fluorescence for the HepG2 cells treated with down‐regulated miRNA‐155 (0.40‐fold). This CLSM result was in accordance with FCM (2.94‐fold and 0.28‐fold, Figure 4H) and RT‐PCR results (2.12‐fold and 0.37‐fold, Figure 4I).

**Figure 5:**
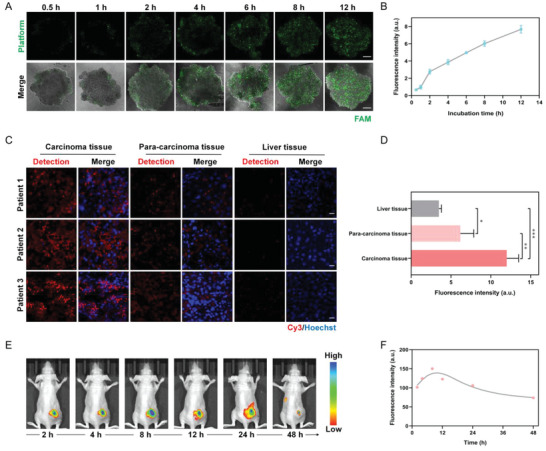
Feasibility of the nano‐theranostic platform for miRNA‐155 imaging in situ hybridization and in living mice. A,B) Penetration of the platform in multicellular HepG2 spheroid models and time‐evolution quantitative analysis (*n* = 3). Scale bars, 100 µm. C,D) The miRNA‐155 imaging in situ hybridization from excised carcinoma, para‐carcinoma, and liver tissue of liver cancer patients, and the corresponding fluorescence intensity (*n* = 3), data are presented as mean ± SD, and significance is determined using ANOVA with post hoc multiple comparisons (LSD). Scale bars, 20 µm. *p < 0.05, **p < 0.01, ***p < 0.001. E,F) Time‐dependent whole‐body fluorescence monitor in living mice and corresponding fluorescence intensity (*n* = 3).

Before miRNA detection in situ hybridization and living animals, the tumor penetration efficiency of the platform was assessed by utilizing a HepG2 tumor spheroid model and monitored via the CLSM. The Z‐stacked images of the center of tumor spheroids treated with our platforms in different periods were depicted (**Figure** **5**A,B) and green fluorescence represented the presence of the platform. The platforms could move closer to the core of tumor spheroids over time. And the tumor spheroids could be covered with the platforms within 12 h. Thus, our platform possessed excellent tumor penetration efficiency to be applicable for in vivo theranostics.

For miRNA detection in situ hybridization of pathologic tissue biopsies, our platform was in situ hybridized with carcinoma tissue, para‐carcinoma tissue, and liver tissue from three liver cancer patients in surgery (Figure 5C and Figure S9, Supporting Information). A strong Cy3 fluorescent signal (red staining) was visualized in carcinoma tissue, a weak signal in para‐carcinoma tissue, and a scarce signal in liver tissue. Fluorescence intensities of carcinoma and para‐carcinoma tissue were 3.44‐fold (*p* < 0.001) and 1.77‐fold (*p* < 0.05) larger than that of the liver tissue, respectively (Figure 5D). And miRNA expression difference between carcinoma tissue and para‐carcinoma tissue was also observed (1.94‐fold, *p* < 0.01). And similar results were observed in carcinoma tissue and liver tissue from tumor‐bearing mice (Figures S10 and S11, Supporting Information, 3.01‐fold, *p* < 0.05). Above all, our platform can achieve miRNA expression detection in cancer tissues in situ hybridization of pathologic tissue biopsies, and distinguish between neoplastic and non‐neoplastic components via measuring miRNA expression level.

For miRNA detection in living mice, the time‐dependent whole‐body fluorescence was monitored after intratumoral injection of the nano‐theranostic platform for BALB/c nude mice with subcutaneous HepG2 tumor (Figure 5E,F). The fluorescence showed an obvious enhancement over time and reached the maximum at 8 h after injection, and then decreased over time. The signal enhancement within 8 h was attributed to the operation of the nano‐theranostic platform, initiated by miRNA‐155 in the tumor region. And at 48 h after injection, the tumor site exhibited very weak fluorescence intensity, signifying an almost complete clearance. These results demonstrated that our platform enabled specific on‐site activation by miRNA‐155 and imaged miRNA‐155 in living mice, manifesting promising potential in advanced molecular imaging of living systems.

### 2.5 Antitumor Efficiency of the Nano‐Theranostic Platform In Vitro

**Figure 6:**
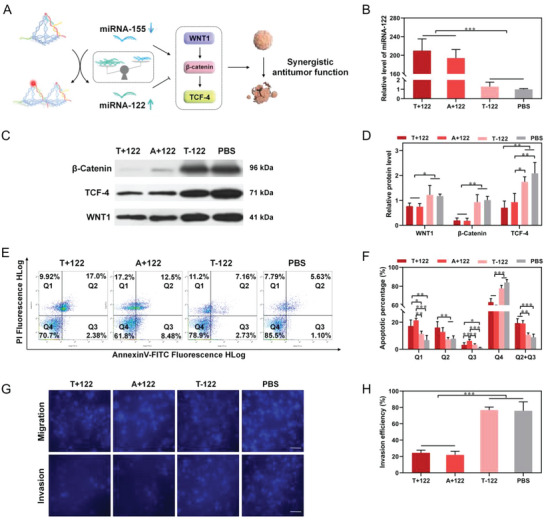
Antitumor efficiency of the nano‐theranostic platform in vitro. A) Schematic illustration of the synergistic regulation of increased miRNA‐122 and decreased miRNA‐155 targeting the Wnt/*β*‐catenin‐TCF signaling pathway. Unbalanced scale: Increased miRNA‐122 and decreased miRNA‐155. B) The relative miRNA‐122 expression levels of four groups (*n* = 3), data are presented as mean ± SD, and significance is determined using ANOVA with post hoc multiple comparisons (LSD). C,D) WB analysis of proteins WNT1, TCF‐4, and *β*‐catenin, and intensities of each protein band (*n* = 3), data are presented as mean ± SD, and significance is determined using ANOVA with post hoc multiple comparisons (LSD). E,F) Cell apoptosis analysis after four treatments for 72 h and corresponding apoptotic percentage (*n* = 3), data are presented as mean ± SD, and significance is determined using ANOVA with post hoc multiple comparisons (LSD). G,H) Migration (upper panel) and invasion (lower panel) analysis after four treatments and corresponding invasion efficiency (*n* = 3), data are presented as mean ± SD, and significance is determined using ANOVA with post hoc multiple comparisons (LSD). Scale bars, 100 µm. *p < 0.05, **p < 0.01, ***p < 0.001.

After verifying that our nano‐theranostic platform can detect miRNA at the cell, tissue, and living body levels, we further explored whether our platform could play an antitumor therapeutic role in living cells. Toward that end, we preloaded the tumor suppressor miRNA‐122 in the platform and delivered them into the HepG2 cells. As depicted in **Figure** **6**A, both miRNA‐122 and miRNA‐155 target the same Wnt/*β*‐catenin‐TCF signaling pathway and regulate the pathway with opposite roles; miRNA‐122 prevents the HCC from progressing by inhibiting this signaling pathway, whereas miRNA‐155 promotes the advancement of HCC. Triggered by the presence of miRNA‐155, the preloaded miRNA‐122 are released; through multiple dual‐miRNAs guided self‐feedback circuits, miRNA‐122 overtly increases while miRNA‐155 decreases inside cells. Thus, these two miRNAs, elevated miRNA‐122 and decreased miRNA‐155, synergistically suppress the proliferation, migration, and invasion of HCC cells and at the same time promote their apoptosis, thereby realizing the therapy.

To further confirm that the therapeutic effect was due to the synergistic regulations of miRNAs rather than the sequences and the structure of our tetrahedral nanovehicles, we divided HepG2 cells into four groups with the following treatments respectively: nano‐theranostic platform with miRNA‐122 (T+122), nano‐theranostic platform without miRNA‐122 (T‐122, as the positive control), miRNA‐122 agomir (a cholesterol‐modified miRNA mimic) (A+122), and PBS. Decreased expression of miRNA‐122 was observed in HCC, and upregulation of miRNA‐122 suppressed the hepatocarcinogenesis or the HCC progression. At first, exogenous delivery of miRNA‐122 to HepG2 cells by our nano‐theranostic platform or with transfection agents were carried out. The expressions of miRNA‐122 after treatments were quantified via RT‐PCR (Figure 6B). The miRNA‐122 expression levels were increased by ≈200‐fold and ≈190‐fold in the T+122‐treated group and A+122‐treated group, respectively, compared to that of the PBS‐treated group. No apparent variations of miRNA‐122 level were observed in T‐122‐ and PBS‐treated groups. These results suggested that the nano‐theranostic platform could be a powerful miRNA delivery system.

After being successfully delivered into cells, elevated miRNA‐122 and decreased miRNA‐155, could regulate the Wnt/*β*‐catenin‐TCF signaling pathway, by binding to the 3’‐UTR of specific mRNAs and triggering mRNA degradation or translational repression. To illuminate the molecular mechanism of our platform for antitumor effect, western blot (WB) revealed a remarkably down‐regulated protein expression of WNT1, *β*‐catenin, and TCF‐4 in both T+122‐ and A+122‐treated groups, in contrast to T‐122‐ and PBS‐treated groups (Figure 6C,D). The WB results indicated that synergistic regulations of dual‐miRNAs suppressed the expression of Wnt/*β*‐catenin‐TCF signaling pathway, which is of significance to carcinogenesis, including proliferation, apoptosis, migration, and invasion of HepG2 cells.

Within 12 h of the above four treatments, all the cell viability stabilized at around 100%. After 12 h treatments, with T+122 and A+122, cell viability significantly decreased, suggesting that the delivered miRNA‐122 via the smart platform inhibited cell proliferation (Figure S12, Supporting Information). To further test the antitumor effects of various treatments, the FCM analysis was applied for apoptosis analysis. Significant increase in the apoptotic percentage was observed in T+122‐ and A+122‐treated groups compared with the other two (T‐122 and PBS) groups, after 48 h (Figure S13A,B, Supporting Information) and 72 h (Figure 6E,F). Clearly, the nano‐theranostic platform preloaded with miRNA‐122 effectually suppressed cell proliferation and promoted their apoptosis, thereby exhibiting strong antitumor capacity.

Furthermore, invasion and migration are also indispensable indicators for evaluating tumor development. Toward that end, we inoculated HepG2 cells having been harvested after four treatments for 48 h into a Transwell chamber, with or without coverage of Matrigel gel for 24 h (Figure 6G). The migration ability can be assessed by the average number of cells migrating through the Transwell chamber without Matrix gel (Figure 6G, upper panel). And the invasion ability can be assessed by the average number of cells migrating through the Transwell chamber with Matrix gel (Figure 6G, lower panel). And the invasion efficiency was calculated by: Invaded cells/migrated cells. The invasion efficiency in T+122 and A+122 treated groups obviously reduced (Figure 6H and Figure S14, Supporting Information), revealing that the platform with miRNA‐122 attenuated migration and invasion of HepG2 cells. Together, the above results collectively demonstrated that the nano‐theranostic platform preloaded with miRNA‐122 effectively transported miRNA‐122 into cells, boosted the expression level of miRNA‐122 and suppressed the proliferation, migration, and invasion of HepG2 cells, thus achieving an excellent antitumor performance in vitro.

### 2.6 Antitumor Efficiency of the Nano‐Theranostic Platform In Vivo

**Figure 7:**
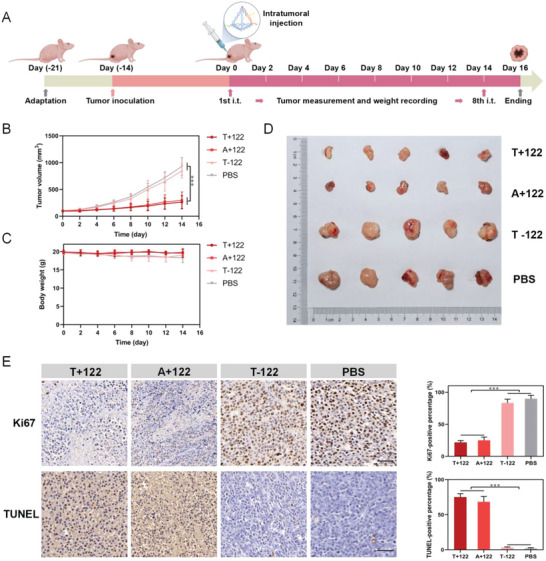
Antitumor efficiency of the nano‐theranostic platform in vivo. A) Schematic illustration of the timeline for the therapeutic experiments via intratumoral injection with four treatments. B,C) Recording of tumor volume and body weight after four treatments (*n* = 5). D) HepG2 tumors excised from tumor‐bearing mice and photographed. E) Immunohistochemistry for Ki67 (upper panel) and TUNEL (lower panel) staining of excised tumors with four treatments and quantitative analysis of positive percentage (*n* = 3), data are presented as mean ± SD, and significance is determined using ANOVA with post hoc multiple comparisons (LSD). Scale bars, 50 µm. ***p < 0.001.

The aforementioned experiments had shown the antitumor effects of the nano‐theranostic platform on HepG2 cells in vitro. To further validate this platform in vivo, we established a HepG2‐tumor‐bearing mice model on the hips of BALB/c nude mice. About 14 days after the xenograft (when the HepG2 tumor volumes reached about 100 mm^3^), we intratumorally injected tumor‐bearing mice with T+122, A+122, T‐122, and PBS once every 2 days for eight times (**Figure** **7**A). The intratumoral injection was adopted due to the high therapeutic index and low toxicity.^[^ ^58^, ^59^ ^]^ The tumor volumes and body weights of the mice were monitored every 2 days after injections. The average tumor volumes of all groups were no more than 1000 mm^3^ in the tumor‐bearing model within treatments for 2 weeks. Tumors grew rapidly in T‐122 and PBS‐treated groups (without miRNA‐122), whereas, the tumor growth of the other two groups (with miRNA‐122) was inhibited (Figure 7B). Results revealed that synergistic regulation of increased miRNA‐122 and relatively decreased miRNA‐155 was indeed able to statistically significantly inhibit tumor growth (*p* < 0.001). Meanwhile, no significant change in body weight during the treatment was observed in any group (Figure 7C). These results suggested a therapeutic effect without biotoxicity. At the end of the treatment, mice were euthanized, and tumors were resected and measured (Figure 7D). Notably, the mice receiving the T+122 treatment displayed tumor growth inhibition, validating the synergistic therapeutic functionality of the platform‐delivered miRNA‐122 and relatively decreased miRNA‐155. To further evaluate the proliferation and apoptosis status of the excised tumors, immunohistochemistry and immunofluorescence for Ki67 and terminal dUTP nick‐end labeling (TUNEL) were performed (Figure 7E and Figure S15, Supporting Information). Ki67 staining showed a marked reduction of Ki67‐positive tumor cells in both T+122‐ and A+122‐treated groups. And TUNEL staining indicated that with treatments of T+122 and A+122, the TUNEL‐positive cells significantly increased compared with the control groups. These results again provided in vivo evidence that T+122 treatment mediated inhibition of cell proliferation and apoptosis induction of the tumor.

We also assessed the in vivo biosafety by quantitatively measuring the hematology and biochemistry of the mice (Figure S16, Supporting Information). No obvious abnormalities were detected in the blood routine examination and kidney functions. Nevertheless, abnormalities were observed in the liver function indicators due to the HepG2 tumor loading. Interestingly, these indicators improved after T+122 and A+122 treatments. The routine histological examinations all showed no obvious lesions in major organs, including hearts, livers, spleens, lungs, and kidneys (Figure S17, Supporting Information), with the structural and functional integrity of the tissues maintaining intact.

## 3 Conclusions

Based on dual‐miRNAs guided self‐feedback tetrahedral entropy‐driven DNA circuit, we have developed a smart nano‐theranostic platform that detects intracellular onco‐miRNAs while at the same time achieves cancer treatment by releasing tumor suppressor miRNAs. Our self‐designed DNA tetrahedron functions as a miRNA loading and delivery vehicle for in vivo applications. And our self‐designed entropy‐driven DNA circuit operates as miRNA in situ amplifiers for ultrasensitive biosensing. Thus, in this report, by delicately programming the four vertexes and six sides of the nano‐theranostic platform, we detected, not only in vitro but also in vivo, oncogenic miRNA‐155 with satisfactory analytical performances.

Meanwhile, by designing this smart nano‐theranostic platform, we preloaded, delivered, and released tumor suppressor miRNA‐122 into cancer cells. Through the triggering of intracellular entropy‐driven DNA circuits and regeneratable self‐feedback, the markedly increased tumor suppressor miRNA‐122 and the relatively decreased onco‐miRNA‐155 synergistically suppressed cancer cell progression including proliferation, migration and invasion. Our platform also promoted apoptosis of cancer cells. In conclusion, our smart nano‐theranostic platform detects onco‐miRNAs and at the same time delivers specific tumor suppressor miRNAs and exerts enhanced antitumor effects both in vitro and in vivo without notable side effects. To our best knowledge, this is the first successful demonstration that combines diagnosis and therapy together via DNA nanotechnology and DNA nanomaterials. We envision that our platform can load other theranostics‐based nucleic acids into the DNA tetrahedral framework and be endocytosed for in vivo theranostics. And our platform will be of value in the clinical setting from early diagnoses to tailored therapies.

## 4 Experimental Section

### Materials

All oligonucleotides (Table S1, Supporting Information), purified via high‐performance liquid chromatography, were obtained from Sangon Biotech Co., Ltd. (Shanghai, China). RPMI 1640 medium and FBS were acquired from VivaCell (Shanghai, China), and penicillin/streptomycin solution and PBS were purchased from Gibco (NY, USA). The SanPrep Column microRNA Extraction Kit, miRNA First Strand cDNA Synthesis kit (Stem‐loop Method), 2X SG Fast qPCR Master Mix kit, and anti‐WNT1 antibody and LipoHigh transfection reagent were purchased from Sangon Biotech Co., Ltd. (Shanghai, China). CCK‐8 Cell Proliferation and Cytotoxicity Assay Kit was bought from Solarbio (Beijing, China). Hoechst33342 was supplied by Invitrogen (MO, USA). The Annexin V‐FITC kit was purchased from Sigma‐Aldrich Co. (MO, USA). The anti‐*β*‐catenin antibody and anti‐TCF‐4 antibody were acquired from Cell Signaling Technology, Inc. (MA, USA) and Abcam (Cambridge, UK). The BioCoat Matrigel Invasion Chambers with 8.0 µm polyester (PET) Membrane, Permeable Support for 24‐well Plate with 8.0 µm Transparent PET Membrane, and Spheroid Microplates were purchased from Corning (NY, USA). HepG2 cells and L‐O2 cells were obtained from the American Type Culture Collection (ATCC, USA). The BALB/c nude mice (*n* = 20, female, 4–6 weeks) were bought from Hunan SJA Laboratory Animal Co., Ltd. (Hunan, China), and animal testing was approved by the Laboratory Animal Welfare and Ethical Committee of Third Medical Military University and operated on the basis of the Guide for the Care and Use of Laboratory Animals (approval number: AMUWEC20223583). The carcinoma tissue, para‐carcinoma tissue, and liver tissue from three liver cancer patients in surgery were collected from the Third Medical Military University Southwest Hospital with approval by the ethics committee of the hospital (approval number: KY2020146). And all participants provided informed, written informed consent.

### Instruments

The fluorescence spectra and the CCK‐8 assay were performed using a Thermo Scientific Varioskan Flash (MA, USA). The fluid mode AFM was carried on NanoWizard BioAFM (Bruker, MA, USA). The DLS was also performed using a Zetasizer Nano ZSP (Malvern, USA). The CLSM imaging was conducted utilizing a ZEISS LSM780 (Zeiss, Germany). The RT‐PCR was analyzed via a CFX96 Real‐Time System (Bio‐Rad, USA). The FCM analysis was performed using a BD FACSCanto II flow cytometer (BD, USA). The in vivo imaging for mice was recorded on IVIS Spectrum CT (PerkinElmer, USA).

### Construction and Characterization of the Nano‐Theranostic Platform

The nano‐theranostic platform was built with a tetrahedral framework and the three‐stranded substrate complex (S). The P3, P5, and P6 (1 µM; 2 µL) were equally mixed into 4 µL of TM buffer (20 mm Tris‐HCl; 50 mm MgCl_2_; pH 8.0) and denatured at 95 °C and slowly cooled down to 4 °C to construct the three‐stranded substrate complex (S). And subsequently, P1, P2, and P4 (1 µM; 2 µL) were added to the above mixture and heated for 10 min at 55 °C, 5 min at 45 °C, 5 min at 37 °C, 5 min at 25 °C, and maintained at 4 °C to architect the tetrahedral framework, thus constructing the platform. The synthesis process of the platform was verified utilizing 5% PAGE and then the constructed platform was scanned on the preconditioned dry mica substrate via fluid mode AFM. The size distribution and zeta potential were recorded using a Zetasizer.

### Analytical Performance for miRNA‐155 Detection In Vitro

Different concentrations of miRNA‐155 from 0 to 100 nM were incubated with the constructed platform for 60 min at 37 °C in the reaction buffer (10 mm Tris‐HCl, 2 mm MgCl_2_, pH = 8.0). And then the measurements of fluorescence spectra were proceeded from 520 to 660 nm at an excitation wavelength of 500 nm. And for specificity assessment, miRNA‐155, various control miRNAs, and a mixture (10 nM) were separately incubated with the platform and measured for fluorescence spectra.

### Reverse Transcription‐Polymerase Chain Reaction Analysis

The miRNAs were extracted from HepG2 cells and L‐O2 cells according to the protocol of the SanPrep Column microRNA extraction Kit. And then, the extracted miRNAs were reverse‐transcribed to complementary DNA using miRNA first strand cDNA synthesis kit, and quantitative PCR was implemented via 2X SG Fast qPCR Master Mix.

### Cellular Uptake Mechanism

The HepG2 cells (10^5^ cells mL^−1^) were plated in 6‐well plates overnight and pretreated at 4 °C or 10 mm M*β*CD (an inhibitor for caveolin‐ dependent endocytosis) or 2 mm AMI (an inhibitor for macropinocytosis) or 10 µg mL^−1^ chlorpromazine (CPZ, an inhibitor for clathrin‐mediated endocytosis) for 30 min, respectively. And then the cells were incubated with the platform (100 nM) for 2 h, and monitored for fluorescence via FCM.

### Colocalization Experiments

The HepG2 cells (10^5^ cells mL^−1^) were plated on the confocal dish for 24 h and incubated with the platform at a final concentration of 100 nM for 0, 0.5, 1, 2, 4, and 6 h at 37 °C. Then, the cells were stained with the LysoTracker (50 nM) for 1 h and stained with Hoechst dye for 10 min. Finally, the cells were monitored for fluorescence using a ZEISS LSM780.

### Confocal Laser Scanning Microscopy Imaging

The HepG2 cells and L‐O2 cells (10^5^ cells mL^−1^) were plated on the confocal dish for 24 h and incubated with the platform at a final concentration of 100 nM for 0.5, 1, 2, 4, 6, and 8 h at 37 °C. Subsequently, the cells were fixed with 4% paraformaldehyde for 15 min and stained with Hoechst dye for 10 min. Finally, the cells were monitored for fluorescence using a ZEISS LSM780. Simultaneously, the HepG2 cells and L‐O2 cells incubated with the platform were verified by FCM and RT‐PCR.

### Transfection of miRNA‐155 Mimic/Inhibitor

The HepG2 cells (5 × 10^5^ cells well^−1^) were plated in 6‐well plates. After the cell density reached 70–90%, the miRNA‐155 mimic (100 nM) and inhibitor (100 nM) were transfected into cells with LipoHigh transfection reagent (5 µL) according to the instruction for 48 h. And the cells were co‐incubated with the platform (100 nM) for 2 h and monitored for fluorescence and verified.

### Multicellular Tumor Spheroid Models and Tissue Penetration Study

The HepG2 cells (5 × 10^3^ cells well^−1^) were plated in the 96‐well spheroid microplates, and incubated at 37 °C in 5% CO_2_. Daily monitoring of spheroid formation and growth could be done every day. When the diameter of the reached 500 µM, the nano‐theranostic platform (100 nM) was added and incubated for different periods (0.5, 1, 2, 4, 6, 8, and 12 h). Finally, the spheroid microplates were observed by a ZEISS LSM780.

### Fluorescence In Situ Hybridization

The obtained tissues were fixed with 4% paraformaldehyde, dehydrated via gradient alcohol treatment, and embedded in paraffine. Then, formalin‐fixed and paraffin‐embedded tissues were sliced and digested with proteinase K (20 µg mL^−1^) for 15 min. And the tissues were incubated with the nano‐theranostic platform (100 nM) at 42 °C overnight and stained with 4’,6‐diamidino‐2‐phenylindole (DAPI) dye. Finally, the FISH was observed by the fluorescence microscope.

### Western Blot

The HepG2 cells (5 × 10^5^ cells well^−1^) were plated in 6‐well plates. After the cell density reached 70–90%, the cells received four treatments for 48 h: (1) T+122 (100 nM; 9.76 µg); (2) A+122 (100 nM); (3) T‐122 (100 nM); (4) PBS. And total proteins were extracted from the treated cells and quantified via the BCA protein assay kit. Then, the proteins were separated by 10% SDS‐PAGE and transferred onto polyvinylidene fluoride membranes. Through standard western blot procedures, the protein levels of Wnt1, *β*‐catenin, and TCF‐4 were quantified via the anti‐WNT1 antibody, anti‐*β*‐catenin antibody, and anti‐TCF‐4 antibody, respectively.

### Cell Proliferation Assay

The HepG2 cells (3 × 10^3^ cells well^−1^) were seeded in 96‐well plates and incubated overnight. After receiving above mentioned four treatments for different periods (1, 2, 4, 8, 12, 24, 48, and 72 h), 10 µL of CCK‐8 was added to the cells and incubated for 4 h, and absorption values were recorded at 450 nm.

### Cell Apoptosis Assay

The HepG2 cells were plated in 6‐well plates (5 × 10^5^ cells well^−1^) and incubated overnight. After four treatments for 48 and 72 h, the cells were incubated with 5 µL of annexin‐V and 1 µL of propidium iodide at room temperature for 15 min. At last, the stained cells were analyzed with FCM.

### Cell Migration and Invasion Assay

The above mentioned‐treated HepG2 cells (5 × 10^4^ cells well^−1^; 0.5 mL) were treated with serum starvation and seeded in the upper chamber of the BioCoat matrigel invasion chambers with 8.0 µm PET membrane with coverage of matrigel (for invasion assay) and permeable support for 24‐well plate with 8.0 µm transparent PET membrane without coverage of matrigel (for migration assay). And RPMI 1640 containing 30% FBS (0.75 mL) was added to the lower chamber, serving as the chemoattractant. After overnight cultivation, the remained cells on the surface of the upper chamber were removed, and the invaded or migrated cells were fixed with 100% methanol and stained with DAPI, and observed with the fluorescence microscope. The invasion efficiency was calculated by invasion efficiency (%) = Average number of cells invading through the Transwell chamber with Matrix gel/Average number of cells migrating through the Transwell chamber without Matrix gel.

### Establishment of Hepatoma Cell Lines‐Tumor‐Bearing Mice Model and Animal Experiment

The HepG2 cells (10^7^ cells) were subcutaneously injected into the hips of the mice treated with isoflurane anesthesia. Tumor growth was measured with a vernier caliper, and the tumor volume was calculated as: a × b^2^/2 (a is the long axis and b is the short axis). And after tumor reached about 100 mm^3^, the mice were randomly divided into four groups, receiving four treatments once every 2 days for eight intratumoral injections: (1) T+122 (10 µM; 100 µL; 97.6 µg); (2) A+122 (10 µM; 100 µL); (3) T‐122 (10 µM; 100 µL); (4) PBS (100 µL). During the treatment, the living status, weight, and tumor volume of mice were recorded. And time‐dependent (2, 4, 8, 12, 24, and 48 h) whole‐body fluorescence was monitored and analyzed by IVIS Spectrum CT for in vivo biodistribution of the nano‐theranostic platform. After 8 treatments, the mice were anesthetized and sacrificed. Blood was collected via eyeball enucleation for hematology and biochemistry. And tumors were excised for the photographic records. And immunofluorescence and immunohistochemistry for Ki67 and TUNEL of the excised tumors were performed to evaluate the proliferation and apoptosis status. Major organs, including the hearts, livers, spleens, lungs, and kidneys of mice were acquired for routine histological examination.

### Statistical Analysis

All data were presented as mean ± standard deviation (SD). The sample size (*n*) for each statistical analysis was noted in each figure legend. Comparison between two groups was performed using independent‐samples T‐tests, and comparison of multiple groups was implemented with one‐way analysis of variance with post hoc multiple comparisons (least significant difference, LSD). The level of statistical significance was indicated on the graphs by asterisks (*, **, or ***) for *p*‐values of less than 0.05, 0.01, and 0.001, respectively. Statistical analyses were conducted via SPSS (version 20.0).

## Conflict of Interest

The authors declare no conflict of interest.

## Supporting information

Supporting Information

## Data Availability

The data that support the findings of this study are openly available in the supplemanentary file of this manuscript.
