# Supplementary material for: A Smart Nano‐Theranostic Platform Based on Dual‐microRNAs Guided Self‐Feedback Tetrahedral Entropy‐Driven DNA Circuit
Source: Adv Sci (Weinh). 2023 Apr 21;10(19):2301814. doi: 10.1002/advs.202301814 (PMC10323617; doi:10.1002/advs.202301814)
Supplement: Supplementary file 1 — Supporting Information [file ADVS-10-2301814-s001.pdf]

ADVANCED SCIENCE
Open Access

Supporting Information

for *Adv. Sci.*, DOI 10.1002/advs.202301814

A Smart Nano-Theranostic Platform Based on Dual-microRNAs Guided Self-Feedback Tetrahedral Entropy-Driven DNA Circuit

Sha Yang, Jie Luo, Ligai Zhang, Liu Feng, Yuan He, Xueping Gao, Shuang Xie, Mingxuan Gao, Dan Luo, Kai Chang and Ming Chen**

Supporting Information

A Smart Nano-theranostic Platform based on Dual-microRNAs Guided Self-Feedback Tetrahedral Entropy-Driven DNA Circuit

Sha Yang[a], Jie Luo[a], Ligai Zhang[a], Liu Feng[a], Yuan He[a], Xueping Gao[a], Shuang Xie[a], Mingxuan Gao[a], Dan Luo[b], Kai Chang[a], Ming Chen[a, c, d*]*

[a] Department of Clinical Laboratory Medicine, Southwest Hospital, Third Military Medical University (Army Medical University), 30 Gaotanyan, Shapingba District, Chongqing 400038, China

[b] Department of Biological and Environmental Engineering, Cornell University, Ithaca NY14853-5701, USA

[c] College of Pharmacy and Laboratory Medicine, Third Military Medical University (Army Medical University), 30 Gaotanyan, Shapingba District, Chongqing 400038, China

[d] State Key Laboratory of Trauma, Burn and Combined Injury, Third Military Medical University (Army Medical University), 30 Gaotanyan, Shapingba District, Chongqing 400038, China

Corresponding Author

* Ming Chen

Tel: +86-23-68766591

Fax: +86-23-68716530

E-mail: chming1971@126.com

* Kai Chang

Tel: +86-23-68754448

Fax: +86-23-68716530

Email: changkai0203@163.com

Biocompatibility of the nano-theranostic platform

A colorimetric Cell Counting Kit-8 (CCK-8) assay was conducted to co-culture the platforms (50 nM and 100 nM) and HepG2 cells for various periods (1, 2, 4, 8, and 12 h). The cell viability was approximately 100% within 12 h, confirming no apparent cytotoxicity or side effects of the nano-theranostic platform over this period (Figure S7, Supporting Information).

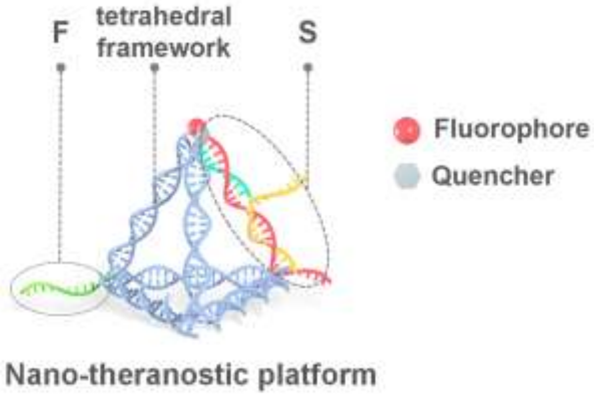

Figure S1. Construction of the nano-theranostic platform.

Sample	Lane 1	Lane 2	Lane 3	Lane 4	Lane 5
Diameter Size (d. nm)	ND	ND	ND	13.89±0.94	14.26±1.24
Zeta Potential (mV)	-1.58±0.58	-2.85±0.35	-4.61±0.51	-6.04±0.79	-7.17±0.67

Figure S2. The changes in diameter sizes and zeta potentials during the assembly process. ND, not determined.

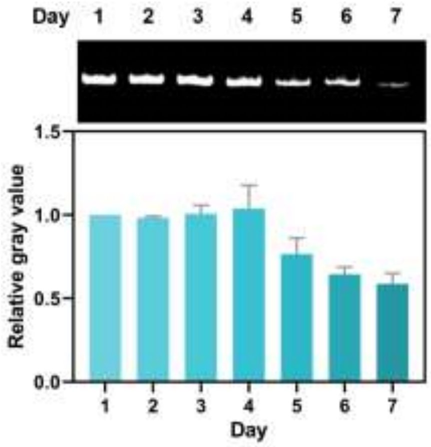

Figure S3. The storage stability of the nano-theranostic platform at 4 °C via native PAGE analysis (n=3).

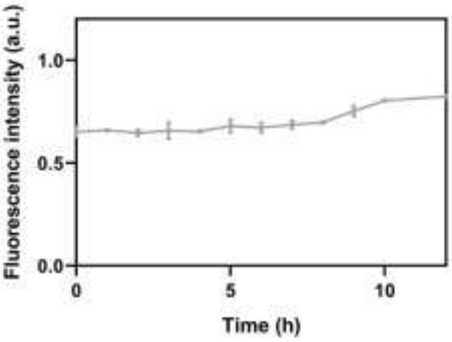

Figure S4. Fluorescence recording of the nano-theranostic platform stability in physiological conditions at different time points (n=3).

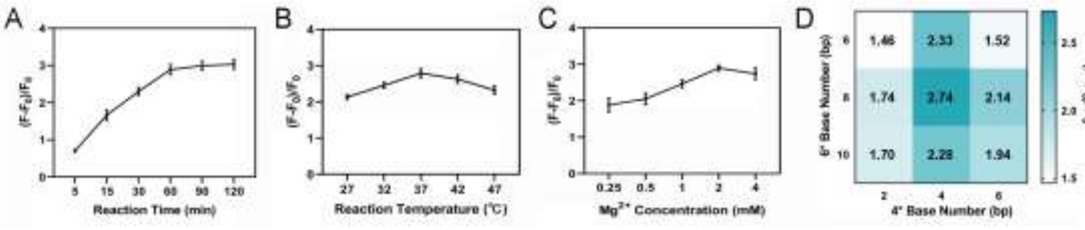

Figure S5. Optimization of experimental conditions (n=3). A) Reaction time. B) Reaction temperature. C) Mg^{2+} concentration. D) Lengths of toehold domains 4 and 6.

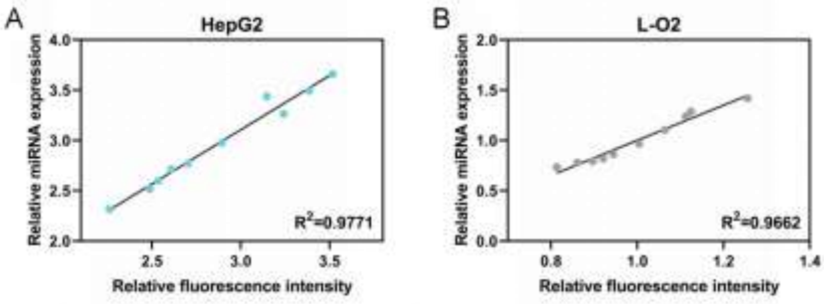

Figure S6. Concordance analysis of our platform and RT-PCR towards miRNA-155 detection in A) HepG2 cells and B) L-O2 cells (n=10).

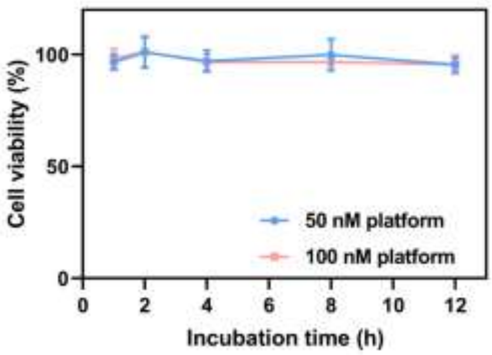

Figure S7. Cytotoxicity of 50 nM and 100 nM platform against HepG2 cells after continuous incubation (n=3).

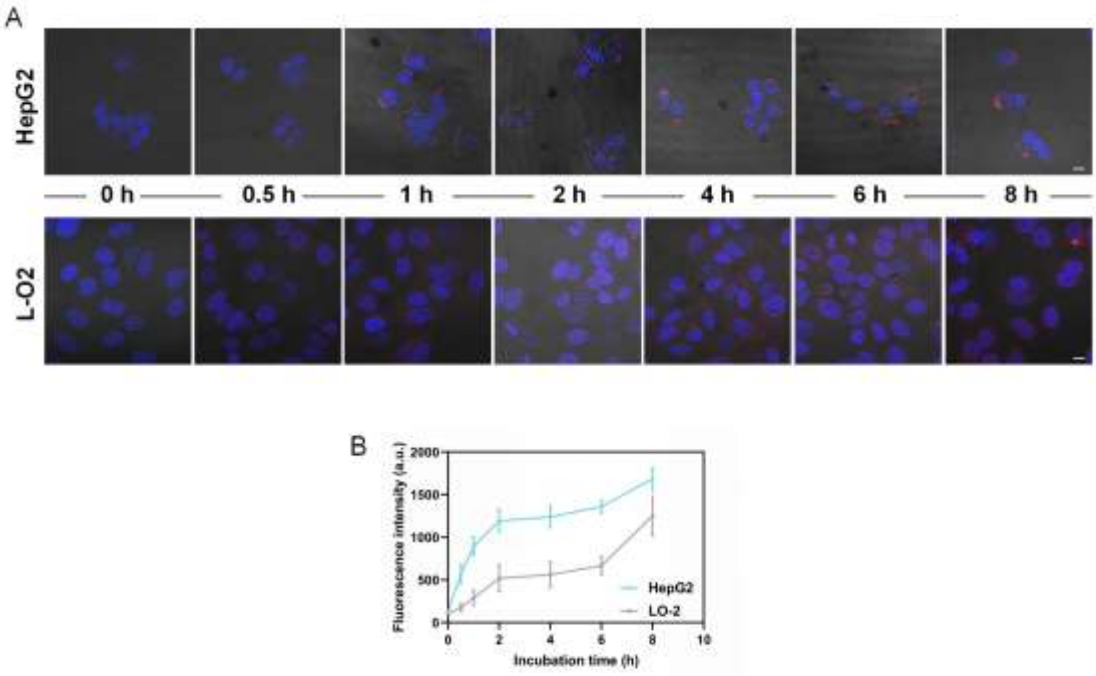

Figure S8. A) CLSM images of HepG2 cells and L-O2 cells incubated with the platform after continuous incubation time. Scale bars, 10 μm. B) Corresponding fluorescence recording (n=3).

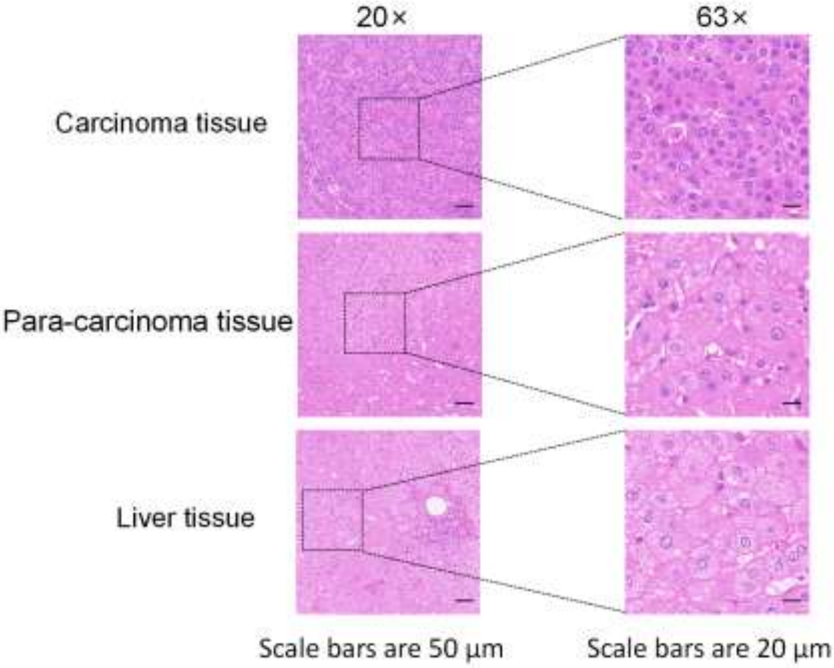

Figure S9. HE staining of carcinoma, para-carcinoma, and liver tissue from liver cancer patients.

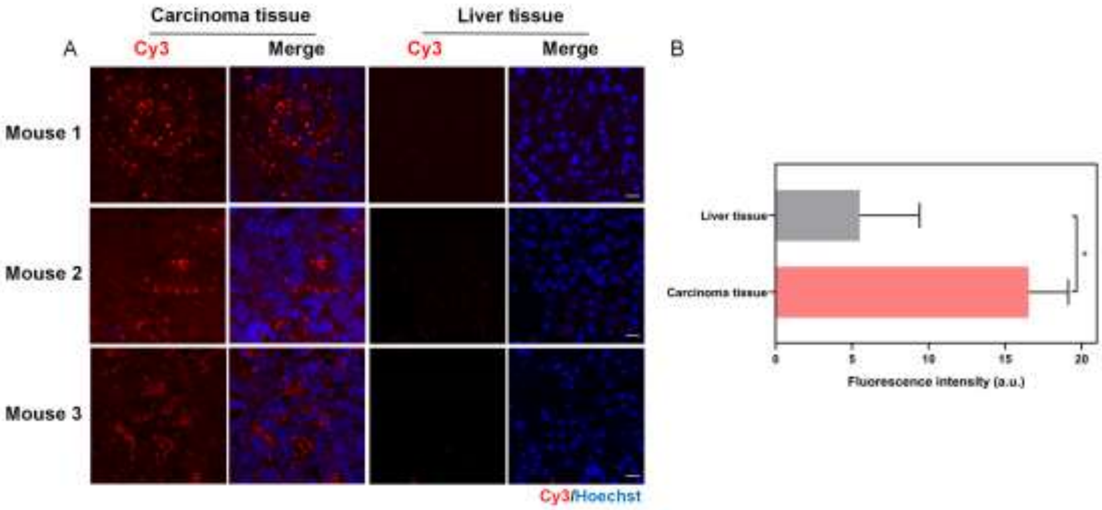

Figure S10. A) The miRNA-155 imaging *in situ* hybridization from excised carcinoma tissue and liver tissue of HepG2 tumor-bearing mice. A) The corresponding fluorescence intensity (n=3), data are presented as mean ± SD, and significance is determined using Independent-Samples T-Tests. Scale bars, 20 μm. **P*

< 0.05.

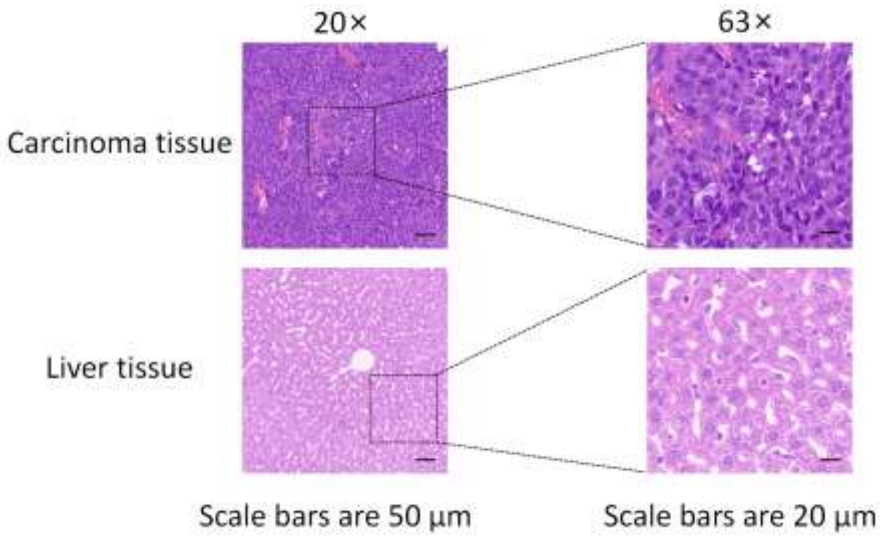

Figure S11. HE staining of carcinoma tissue and liver tissue from HepG2 tumor-bearing mice.

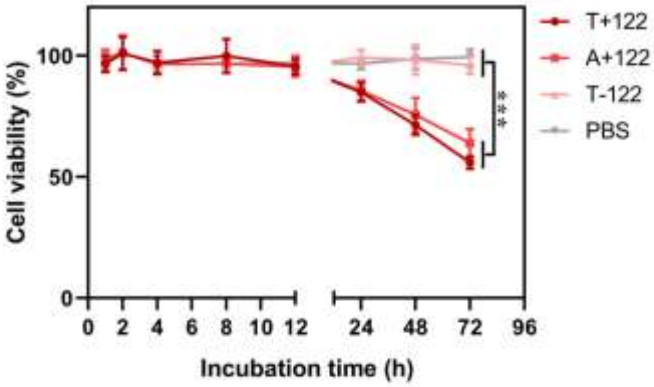

Figure S12. Cytotoxicity of four treatments against HepG2 cells after continuous incubation time (n=3), data are presented as mean ± SD, and significance is determined using ANOVA with Post Hoc Multiple Comparisons (LSD). ***P < 0.001.

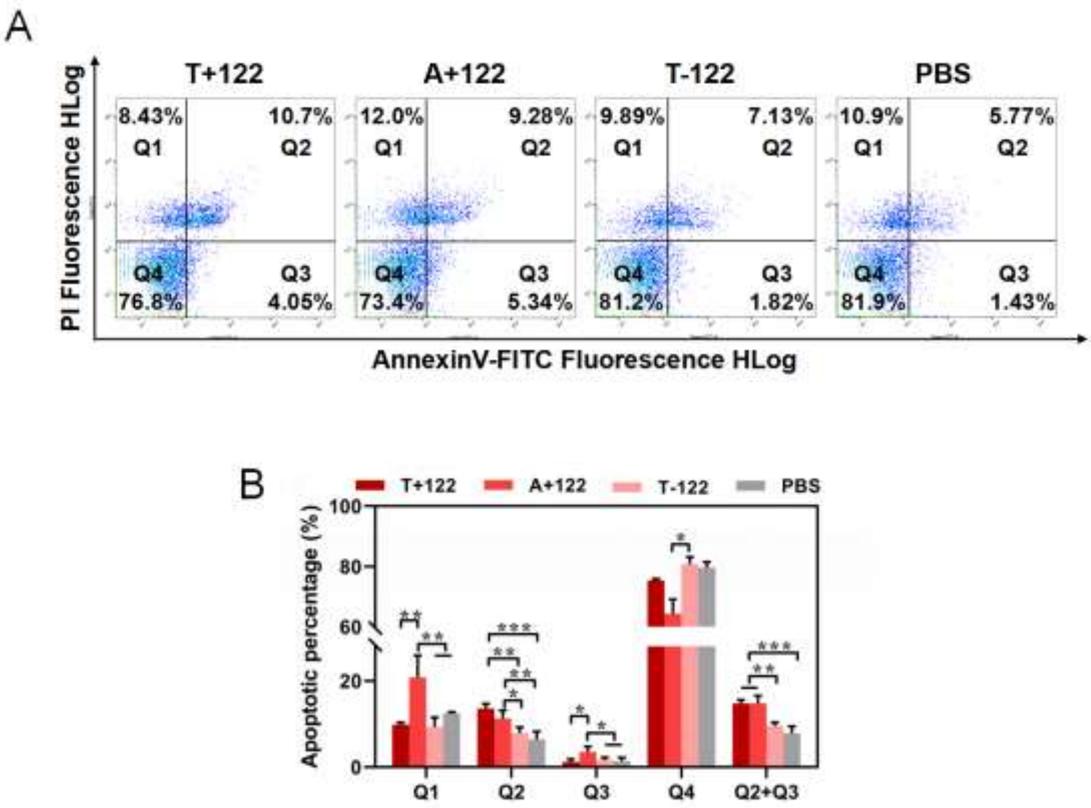

Figure S13. A) Cell apoptosis analysis after four treatments for 48 h and B) corresponding apoptotic percentage (n=3), data are presented as mean ± SD, and significance is determined using ANOVA with Post Hoc Multiple Comparisons (LSD). **P* < 0.05, ***P* < 0.01, ****P* < 0.001.

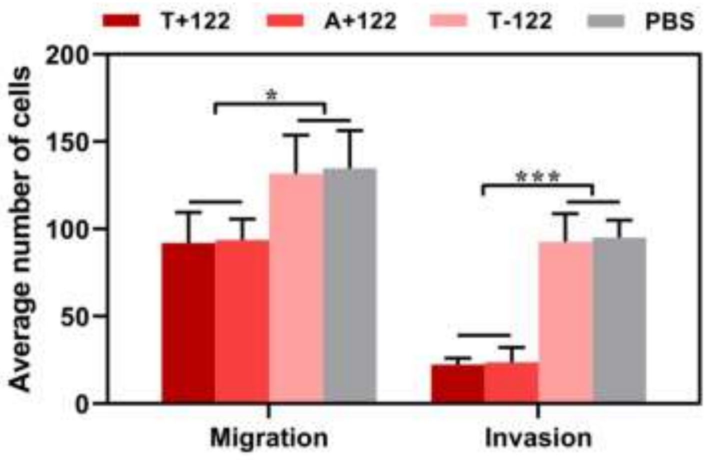

Figure S14. The average number of migrated and invaded cells after four treatments

(n=3), data are presented as mean ± SD, and significance is determined using ANOVA with Post Hoc Multiple Comparisons (LSD). *P < 0.05, ***P < 0.001.

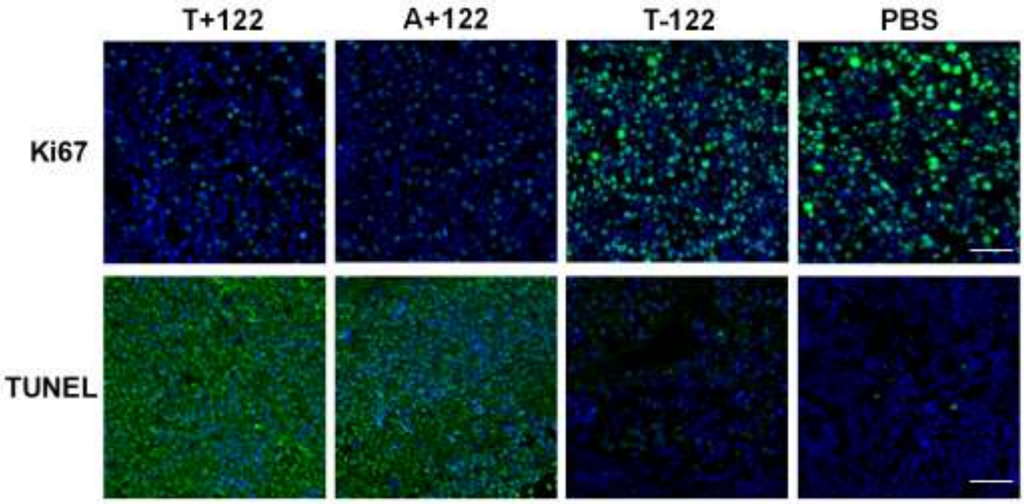

Figure S15. Immunofluorescence for Ki67 (upper panel) and TUNEL (lower panel) staining of excised tumors with four treatments. Scale bars, 50 µm.

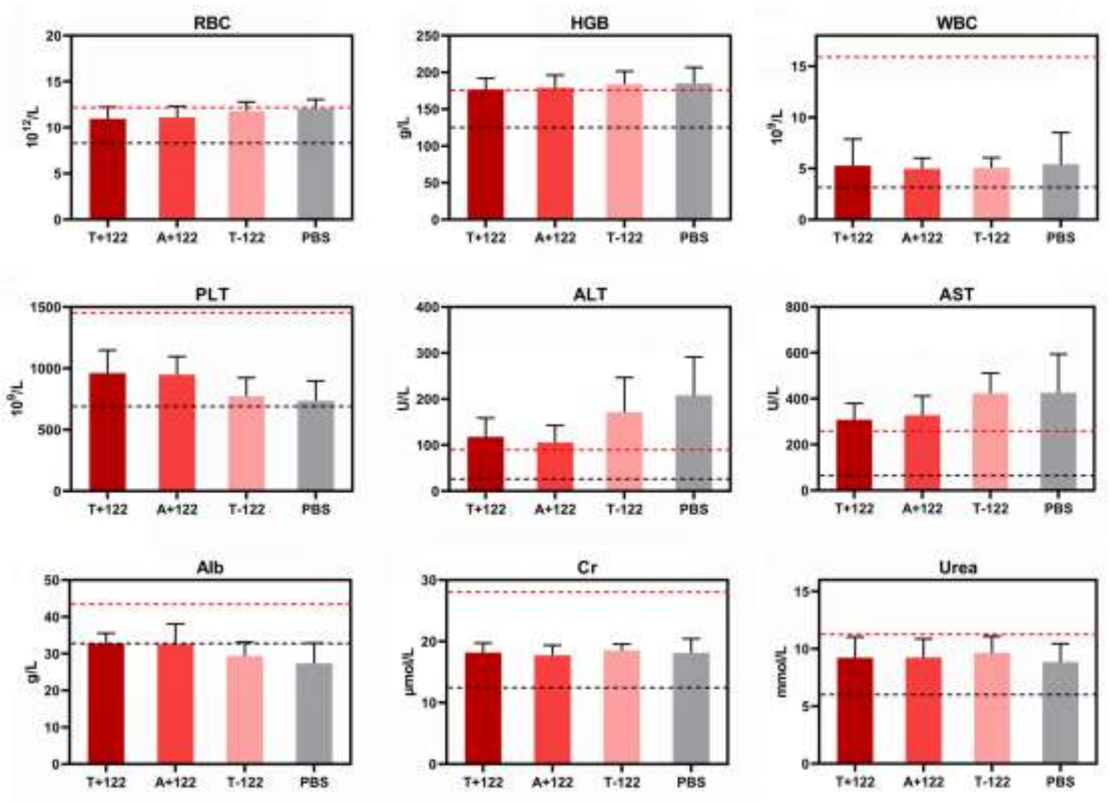

Figure S16. The hematology and biochemistry of tumor-bearing mice with four treatments at the end of the experiment (n=3), data are presented as mean ± SD. Hematology indicators: red blood cell (RBC), hemoglobin (HGB), white blood cell (WBC), and platelet (PLT). Liver function indicators: alanine transaminase (ALT), aspartate transaminase (AST), and albumin (Alb). Kidney function indicators: Cr (creatinine) and Urea.

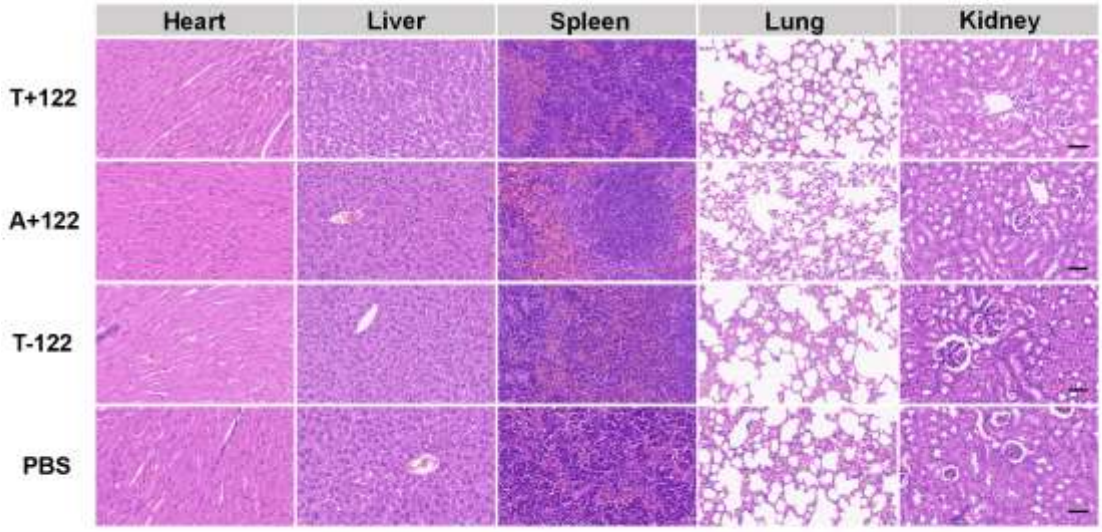

Figure S17. Routine histological examination of excised hearts, livers, spleens, lungs, and kidneys. Scale bars, 50 µm.

Table S1. DNA sequences and modifications

Oligonucleotides	Sequence (5’→3’)
P1	TGGTGGAGGGCGTGTCCTTCTAATAGGGTTTCCTGGTCAAGCGGTGGATGCAAACATCCCTTTTTGGGCTGTTCCGGGTGTGGCTCGTCCG
P2	FAM-TATCACCAGGCAGTTGACAGTGTAGCAAGCTGTAATAG
P3	TTCAGACTTAGGAATGTGCTTCCCACGTAGTGTCGTTT-Cy3-CAAACACCATTGTCACACTCCAAGCAACCCCTATCACGATTAGCATTAA
P4	TCAACTGCCTGGTGATAAAACGACACTACGTGGGAATCTACTATGGCGGCTCTTC
miRNA-122 (12)	UGGAGUGUGACAAUGGUGUUUG-BHQ2
Waste (345)	AATCGTGATAGGGGTTGCTTTTTT
miRNA-155 (56)	UUAAUGCUAAUCGUGAUAGGGGU
SMT	UUAAUGCUAAUCGUGAUCGGGGU
DMT	UUAAUGCUAAUCGUGGUCGGGGU
TMT	UUAAUGCUAAUCGUGGUCGGGAU
miRNA-155 inhibitor	(mA)(mA)(mC)(mC)(mC)(mC)(mU)(mA)(mU)(mC)(mA)(mC)(mG)(mA)(mU)(mU)(mA)(mG)(mC)(mA)(mU)(mU)(mA)(mA)
miRNA-21	UAGCUUAUCAGACUGAUGUUGA
miRNA-155-F	AAGCGCCTTTAATGCTAATCGT
miRNA-155-R	CAGTGCAGGGTCCGAGGT
miRNA-155-RT	GTCGTATCCAGTGCAGGGTCCGAGGTATTCGCACTGGATACGACAACCCC
miRNA-122-F	AACACGCTGGAGTGTGACAA
miRNA-122-R	CAGTGCAGGGTCCGAGGT
miRNA-122-RT	GTCGTATCCAGTGCAGGGTCCGAGGTATTCGCACTGGATACGACCAAACA
U6-F	AGAGAAGATTAGCATGGCCCCTG
U6-R	ATCCAGTGCAGGGTCCGAGG
U6-RT	GTCGTATCCAGTGCAGGGTCCGAGGTATTCGCACTGGATACGACAAAATA

Note: underline denotes mismatched base.

Table S2. Relative standard deviation (RSD) of Figure S5

Fig. numbers	Time/ Temperature Concentration/ Base Number	Relative standard deviation (RSD, %)
Figure S5A Reaction Time (min)	5	4.39
	15	8.57
	30	4.69
	60	4.74
	90	4.11
	120	4.27
Figure S5B Reaction Temperature (°C)	27	2.88
	32	3.18
	37	4.44
	42	4.26
	47	4.71
Figure S5C Mg^{2+} Concentration (°C)	0.25	9.98
	0.5	7.72
	1	4.25
	2	2.71
	4	5.60
Figure S5D 4^*/ 6^* Base Number (bp)	2/6	5.02
	4/6	1.92
	6/6	3.76
	2/8	17.49
	4/8	7.58
	6/8	6.16
	2/10	6.62
	4/10	7.94
	6/10	17.60

Table S3. Comparison with other methods for intracellular miRNA detection in the last three years

Method	Detection Range	Orders of magnitude	Lod	Theranostic	Ref
SERS-Fluorescence	0.2 to 2 nM	2	11.8 pM	No	[1]
Fluorescence	0 to 100 nM	3	10 pM	No	[2]
Fluorescence	0.05 to 2 nM	3	21 pM	No	[3]
Fluorescence	0.8 to 100 nM	4	0.72 nM	No	[4]
Fluorescence	1 pM to 1 nM	4	0.77 pM	No	[5]
Fluorescence	0 to 50 nM	2	1.499 nM	No	[6]
Fluorescence	0.5 to 10 μM	3	2.11 nM	Yes	[7]
Fluorescence	0 to 10 nM	2	42.3 pM	Yes	[8]
Fluorescence	1 pM to 10 nM	5	114.62 fM	Yes	This work

Lod: limit of detection. SERS: surface-enhanced Raman scattering.

Table S4. Relative standard deviation (RSD) of Figure 3D

Concentration (LgC / fM)	Relative standard deviation (RSD, %)
Blank	11.08
2	10.41
3	5.45
4	4.02
5	4.20
6	0.88
7	2.62
8	2.09

Table S5. Relative standard deviation (RSD) of Figure 3E

Samples	Relative standard deviation (RSD, %)
Blank	11.08
miRNA-155	2.62
Mixture	4.36
SMT	5.56
DMT	3.02
TMT	4.57
miRNA-122	4.81
miRNA-21	0.63

References

[1] J. Wang, J. Fu, H. Chen, A. Wang, Y. Ma, H. Yan, Y. Li, D. Yu, F. Gao, S. Li, *Biosens. Bioelectron.* **2023**, *224*, 115051.

[2] C. Xue, M. Luo, L. Wang, C. Li, S. Hu, X. Yu, P. Yuan, Z. S. Wu, *Anal. Chem.* **2021**, *93*, 9869.

[3] C. Xing, Q. Lin, X. Gao, T. Cao, J. Chen, J. Liu, Y. Lin, J. Wang, C. Lu, *ACS Appl Mater Interfaces* **2022**, *14*, 39866.

[4] X. Deng, X. Liu, S. Wu, S. Zang, X. Lin, Y. Zhao, C. Duan, *ACS Appl. Mater. Inter.* **2021**, *13*, 45214.

[5] L. Yu, S. Yang, Z. Liu, X. Qiu, X. Tang, S. Zhao, H. Xu, M. Gao, J. Bao, L. Zhang, D. Luo, K. Chang, M. Chen, *Materials Today Bio* **2022**, *15*, 100276.

[6] L. Zhou, M. Gao, W. Fu, Y. Wang, D. Luo, K. Chang, M. Chen, *Sci Adv* **2020**, *6*,

b695.

[7] Y. Gong, W. Yuan, X. Guo, Q. Zhang, P. Zhang, C. Ding, *Mikrochim Acta* **2021**, *188*, 253.

[8] S. Cheng, Y. Shi, C. Su, Y. Li, X. Zhang, *Biosens. Bioelectron.* **2022**, *214*, 114550.
